# Climate Change and Respiratory Diseases: Relationship between SARS and Climatic Parameters and Impact of Climate Change on the Geographical Distribution of SARS in Iran

**DOI:** 10.3390/arm90050048

**Published:** 2022-08-29

**Authors:** Giti Bahrami, Hassan Rafiey, Alireza Shakiba, Mehdi Noroozi, Homeira Sajjadi, Hamed Seddighi

**Affiliations:** 1 Social Determinants of Health Research Center, Alborz University of Medical Sciences, Karaj 3149779453, Iran; 2 Social Welfare Management Research Center, University of Social Welfare and Rehabilitation Sciences, Tehran 1985713834, Iran; 3 Center for Remote Sensing & GIS Research, Faculty of Earth Sciences, Shahid Beheshti University, Tehran 1983969411, Iran; 4 Social Determinants of Health Research Center, University of Social Welfare and Rehabilitation Sciences, Tehran 1985713834, Iran; 5 Department of Social Welfare Management, University of Social Welfare and Rehabilitation Sciences, Tehran 1985713834, Iran; 6 Department of Governance and Innovation, Campus Fryslân, University of Groningen, 8911 CE Leeuwarden, The Netherlands

**Keywords:** severe acute respiratory syndrome (SARS), climate change, neural network, bioclimatic variables, CPRs scenario, temperature, perception, Iran

## Abstract

**Highlights:**

- This is the first study on the effects of climate change on SARS in Iran.
- The result shows a positive association between the incidence of SARS and bio-climatic variables.
- This study shows the hospital admissions for climate-related respiratory diseases in Iran will increase by 36% in 2020–2050.
- The geographical distribution of the SARS will also change in 2050 in Iran.

**Abstract:**

Climate change affects human health, and severe acute respiratory syndrome (SARS) incidence is one of the health impacts of climate change. This study is a retrospective cohort study. Data have been collected from the Iranian Ministry of Health and Medical Education between 17 February 2016 and17 February 2018. The Neural Network Model has been used to predict SARS infection. Based on the results of the multivariate Poisson regression and the analysis of the coexistence of the variables, the minimum daily temperature was positively associated with the risk of SARS in men and women. The risk of SARS has increased in women and men with increasing daily rainfall. According to the result, by changes in bioclimatic parameters, the number of SARS patients will be increased in cities of Iran. Our study has shown a significant relationship between SARS and the climatic variables by the type of climate and gender. The estimates suggest that hospital admissions for climate-related respiratory diseases in Iran will increase by 36% from 2020 to 2050. This study demonstrates one of the health impacts of climate change. Policymakers can control the risks of climate change by mitigation and adaptation strategists.

## 1. Introduction

Climate change can affect human health through an increase in temperature and the amount of precipitation [1,2,3,4,5,6,7,8,9,10,11,12,13]; for instance, floods and disasters triggered by natural hazards can increase the risk of water- and vector-borne diseases [14,15,16,17,18].

However, there have seldom been large-scale, systematic efforts to quantify the human health impacts due to climate change. This original research uses empirical data from 732 locations in 43 countries to estimate the mortality burdens associated with the additional heat exposure that has resulted from recent human-induced warming, during the period of 1991–2018. The overall estimate is 37.0% but this percentage varied widely across sub-regions and countries. The largest climate change-induced contributions (>50%) were in southern and western Asia including Iran [19].

Severe acute respiratory syndrome (SARS) is a viral respiratory disease caused by a SARS-associated coronavirus. It was first identified at the end of February 2003 during an outbreak that emerged in China and spread to four other countries. In this study, patients with SARS were defined as patients with a body temperature of 100.5 F (38 °C) or higher, a dry cough, shortness of breath, and the need for hospitalization. The World Health Organization (WHO) coordinated the international investigation with the assistance of the Global Outbreak Alert and Response Network (GOARN) and worked closely with health authorities in affected countries to provide epidemiological, clinical, and logistical support and bring the outbreak under control. Furthermore, one of the consequences of climate change on human health is respiratory disorders. Severe acute respiratory syndrome (SARS) is the highest second risk for morbidity and the third highest risk for mortality among all age groups in the world [20]. Likewise, studies have shown a relationship between ambient temperature and mortality due to respiratory diseases [21].

Studies indicated that the prevalence of respiratory diseases has markedly increased [22]. Anderson et al. (2013) found a 3.4% increase in the hospitalization rate as a result of SARS infections when the temperature is increased by 10 F per day in America [23]. In South Africa, one study demonstrated the impact of temperature and precipitation on the incidence of SARS among children (a 37.9% increase) [24]. Another study has shown the correlation of SARS prevalence with the mean temperature (*p* = 0.025, β = 0.399), duration of sunshine (*p* = −0.007, β= 0.293), and air pollution (*p* = −0.018, β = 0.079) in Brazil [25]. Moreover, scientific research in New Zealand has found an association between the mortality of individuals with SARS and the number of cold and dry days [26]. Zhang et al. (2019) have shown that heatwaves exert adverse effects on acute upper respiratory infection (Lag 0: RR = 1.306, 95% CI: 1.177–1.450), especially in those 4–17 years old and older. Furthermore, the risk ratio (RR) of outpatient visits for respiratory disorders was statistically significant in females (Lag 0: RR = 1.161, 95% CI: 1.046–1.298), males (Lag 4: RR = 1.161, 95% CI: 1.096–1.261), young individuals with an age range of 4–17 years old (Lag 0: RR = 1.741, 95% CI: 1.524–1.990), and elderly individuals who were 65 years or older (Lag 5: RR = 1.412, 95% CI: 1.111–1.794) during heatwaves [27]. In Denmark, it has been shown that the maximum apparent temperature (Tapp_max_) is significantly correlated with the number of cases of respiratory infection in elderly individuals, as with an 8 °C increase in the 5-day cumulative average of Tapp_max_, a 7% (95% CI: 1%, 13%) increase was found in the respiratory admission rate in the warm period [28].

Gouya et al. (2016) investigated the burden of SARS, namely the trends of SARS over 12 months (seasonality), and the age groups at risk in a number of provinces of Iran. They showed the trend of the incidence of SARS follows a sinusoidal pattern in cold provinces, with a downward trend and a minimum during the warm months, from June to September, while it shows an upward trend during the cold season, from October to May [29].

The association of SARS with climatic factors in Iran has remained unclear despite the vulnerability of Iran to climate change. The present research aimed to assess the relationship between climatic factors and SARS infection in both genders of different ages i different climate zones of Iran. While the majority of studies have focused on climate and its association with the risk of SARS, predicting the prevalence of SARS can also be estimated by the Emission Scenario.

The Intergovernmental Panel on Climate Change (IPCC) established a new set of emission scenarios in 1996 to provide input for the IPCC 3rd assessment (SRES; Special Report of Emission Scenario). One of the Emission Scenarios is information about socioeconomic conditions and the amount of greenhouse emission in the atmosphere, also called the non-climate scenario. There are some factors involved in the increase in greenhouse gasses, such as land-use change and the increased activity of industries and factories for economic development. Therefore, Emission Scenarios were established to gain an overview of the future developments in the global environment and provide a special reference for the emission of greenhouse gasses and particulates in the atmosphere [30].

The IPCC issued new reports of scenarios, termed Representative Concentration Pathways (RCPs). These are scenarios that include time series of emissions and concentrations of the full suite of greenhouse gasses (GHGs) and aerosols and chemically active gasses, as well as land use/land cover (Moss et al., 2008). The word representative signifies that each RCP provides only one of many possible scenarios that would lead to the specific radiative forcing characteristics. The term pathway emphasizes that not only the long-term concentration levels are of interest, but also the trajectory taken over time to reach that outcome (Moss et al., 2010). WGIII). Specifically, these include RCP 2.6, RCP 4.5, RCP 6.0, and RCP 8.5, which are all categorized into four levels from RCP 2.6 to RCP 8.5. Based on this type of classification, the levels of scenario can range from optimistic (RCP 2.6) to pessimistic conditions (RCP 8.5) [30].

In accordance with the reports of WHO (World Health Organization), between 1900 and 2100, the temperature will increase, and the sea levels will rise in Iran [31]. Considering that Iran is a country vulnerable to climate change, our aim in this study is to investigate effects of the climate change on human health, especially the development of SARS. The identification of the impact of the climate change on health could be useful for risk management in social and health policy sectors. In this study, we employed the RCP 4.5 Emission Scenario to predict the impact of climate change on SARS infection in Iran.

The current study includes two parts: The first part describes the relationship between SARS and climatic parameters from 2015 to 2018, and in part 2, the impact of climate change on the geographical distribution of SARS in Iran was evaluated.

## 2. Methods

Part 1: The present retrospective cohort study was conducted using secondary and ecological data on SARS, including the climate type and gender of residents, which were collected from the Iranian Ministry of Health and Medical Education between 17 February 2016 and17 February 2018.

**Figure 1:**
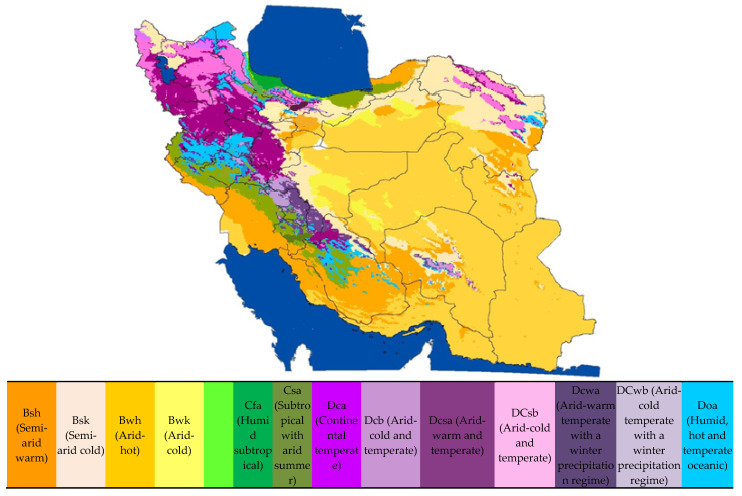
Type of climate in Iran based on Köppen climate classification (A Study of the Impact of Climate Change and Drought Management, a national study, Dr shakiba et al., 2016). For more information you can see: https://en.wikipedia.org/wiki/K%C3%B6ppen_climate_classification and http://koeppen-geiger.vu-wien.ac.at/ (accessed on 8 August 2021). Reprinted from: The relationship between dysentery and climatic parameters in Iran, vol. 34, Giti Bahrami, Mehdi Noroozi, Alireza Shakiba, Hassan Rafiey, HomeiraSajjadi, Pages No.: 100,697, Copyright (2022), with permission from Elsevier [32].

The study area included 169 cities in Iran, where SARS was reported between 17 February 2016 and 17 February 2018. The cities were classified by their climatic characteristics and zone (Master Plan of investigating the effects of climate change and drought management, Supreme Council of Science and Technology Researches of Iran; Reproduced from Supreme Council of Science and Technology Researches of Iran with permission from the Dr.Shakiba) (Figure 1).

Table 1 presents the code and type of each climate, the distribution percentage of climates, and the number and percentage of SARS cases by climate region in Iran.

Meteorological data collected between February 2016 and February 2018 consisted of the maximum temperature, minimum temperature, and daily precipitation obtained from the meteorological organization of Iran (Table 2).

The number of patients with SARS was defined by patients with a body temperature of 100.5 F (38 °C) or higher, a dry cough, shortness of breath, and the need for hospitalization. Of note, the related data were recorded on a daily basis. Other variables were considered independent variables or predictors. Table 2 displays information on the time interval of measuring each variable.

The quantitative variables were analyzed using the mean values, while the categorical variables were expressed as the frequency and relative frequency.

Given the quantitative nature of SARS, as a dependent variable in data analysis, the analysis of the Poisson regression model was utilized for the longitudinal data, and the daily maximum temperature, daily minimum temperature, and daily precipitation were regarded as predictor variables in the bivariate Poisson regression model.

The variables with *p* < 0.2 were then entered into the final model to modify the effects of the other variables. Each analysis was performed for different gender and climate categories. For each variable, the effect size was calculated based on the IRR index (relative risk) and reported with a confidence interval of 95%.

**Poisson regression model**: The time series data, including frequency, were analyzed by modeling the underlying mechanism as a Poisson process. According to the Poisson regression model:

$$E\;\left(y_{t}\;l\;x_{t}\right)=\exp\;(\beta_{0}+\overset{n}{\underset{i=0}{\sum }}\beta_{i}x_{ti}\;)$$

In this equation, *x_t_* represents the vector of the independent variables on day *t* with regression coefficients of *β* and *y_t_* and the dependent variable on day *t*. Estimating the relative risk (RR) as *RRi* = exp(*βi*) constitutes the usefulness of the Poisson regression in climate change, where *βi* represents the regression coefficient associated with a unit increment in the climatic variables. All the data were analyzed in STATA 14.

Part 2: The present study’s second part is a secondary analysis using the Neural Network Model. In this study, based on the reports of SARS, 169 cities of Iran were analyzed between 17 February 2016 and 17 February 2018. Bioclimatic variables were applied as independent variables. Firstly, raster data were downloaded from the WorldClim website (https://www.worldclim.org/ accessed on 13 June 2021); then, their autocorrelation was checked. The collected raster data consisted of information about 19 bioclimatic variables, from the entire earth, in the present and future (2050).

Notably, each bioclimatic variable comprises 19 global climate models (GCMs), and the mean values of GCMs were utilized for each bioclimatic variable (Box 1).

Box 1 **Bioclimatic variables.**

BIO1 = Annual Mean Temperature

BIO2 = Mean Diurnal Range (Mean of monthly (max temp–min temp))

BIO3 = Isothermality (BIO2/BIO7) (×100)

BIO4 = Temperature Seasonality (standard deviation × 100)

BIO5 = Max Temperature of Warmest Month

BIO6 = Min Temperature of Coldest Month

BIO7 = Temperature Annual Range (BIO5–BIO6)

BIO8 = Mean Temperature of Wettest Quarter

BIO9 = Mean Temperature of Driest Quarter

BIO10 = Mean Temperature of Warmest Quarter

BIO11 = Mean Temperature of Coldest Quarter

BIO12 = Annual Precipitation

BIO13 = Precipitation of Wettest Month

BIO14 = Precipitation of Driest Month

BIO15 = Precipitation Seasonality (Coefficient of Variation)

BIO16 = Precipitation of Wettest Quarter

BIO17 = Precipitation of Driest Quarter

BIO18 = Precipitation of Warmest Quarter

BIO19 = Precipitation of Coldest Quarter

In part 2, the Neural Network Model was employed for the prediction of SARS infection. In the present research, a generalized feed-forward type of neural network (GFNN), which is a specific type of the neural network family, was used to predict the SARS infection distribution. For the presently developed neural network, bioclimatic variables were used as input for predicting the SARS distribution. A feed-forward methodology is able to estimate any arbitrary function between the input and output vectors by means of the known input–output relationship of the past, called ‘‘training data’’. The feed-forward approach could also be used for multidimensional interpolation [33]. The basic architecture of the neural network was used in this work. The current network has three network inputs (bio 2, bio 12, and bio 14) and one network output (mean SARS infection per population).

When the network is run, each hidden layer unit performs the calculation in the following equation on its inputs and transfers the result to the next layer of units. This equation is called the activation function’. Even though other types of activation functions exist, the activation function used in the presently developed network is the hyperbolic tangent axon (TanhAxon) and it is given by:

$$f\;\left(x_{i\;}\;w_{i}\right)=tanhx_{i}^{lin}$$

where $x_{i}^{lin}$= $\beta x_{i\;}\;$is the scaled and offset activity inherited from a linear axon that has slope and offset control [34]. Both the root mean square error (RMSE) and the mean absolute error (MAE) are regularly employed in model evaluation studies. Willmott and Matsuura (2005) suggested that the RMSE is not a good indicator of average model performance and might be a misleading indicator of average error, and thus the MAE would be a better metric for that purpose. While some concerns over using RMSE raised by Willmott and Matsuura (2005) and Willmott et al. (2009) are valid, the proposed avoidance of RMSE in favor of MAE is not the solution. Citing the aforementioned papers, many researchers chose MAE over RMSE to present their model evaluation statistics when presenting or adding the RMSE measures could be more beneficial [35]. In the present GFNN, MAE and RMSE have been reported (Table 3).

## 3. Results

Part1: The relationship between SARS and climatic parameters.

SARS was reported in 9220 patients from February 2016 to 2018, including 4241 men (45.99%) and 4979 women (54%). The highest incidence was observed in a warm semi-arid climate (24.84%), and such an incidence was reported to be 45% (4979 cases) in women (Table 1).

### 3.1. Gender-Based Results

The Poisson regression model was utilized as a time series test to determine the relationships between meteorological variables and the risk of SARS with regard to gender. According to Table 4 showing the results of the multivariate Poisson regression model and analysis of the coexistence of variables in men, the minimum temperature (IRR = 0.98, 95% CI:0.98, 0.99, *p* < 0.001) is a protective factor against SARS incidence, whereas no significant relationship was found between climate variables and SARS incidence in women.

### 3.2. Results Based on Climate

Table 5 presents the relationship between SARS and climatic variables by the type of climate and gender. The results are categorized by climate type as follows.

#### 3.2.1. Semi-Arid Cold Climate (BSK)

The minimum daily temperature was positively associated with the risk of SARS in men (IRR = 1.01, 95% CI: 1.00, 1.03, *p* = 0.010) and women (IRR = 1.01, 95% CI: 1.00, 1.02, *p* = 0.004) in a way that the risk is increased by 1% in line with a 1 °C increase in the minimum daily temperature. The risk of SARS is also increased by 1.02% in women (IRR = 1.02, 95% CI: 1.00, 1.05, *p* < 0.000) and 1.03% in men (IRR = 1.03, 95% CI: 1.00, 1.05, *p* = 0.003) with a 1 mL increase in daily rainfall.

#### 3.2.2. Arid-Hot Climate (BWH)

The risk of SARS is decreased in men by 1% with a 1 °C increase in the minimum temperature and 1% in women with a 1 °C increase in the maximum temperature.

#### 3.2.3. Humid, Hot, and Temperate Oceanic Climate (Doa)

The risk of SARS is also decreased by 0.05% in men (IRR = 0.95, 95% CI: 0.92, 0.98, *p* < 0.009) with a 1 mL increase in daily rainfall (Table 5).

Part 2: Prediction of the impact of climate changes on the geographical distribution of SARS in Iran.

**Figure 2:**
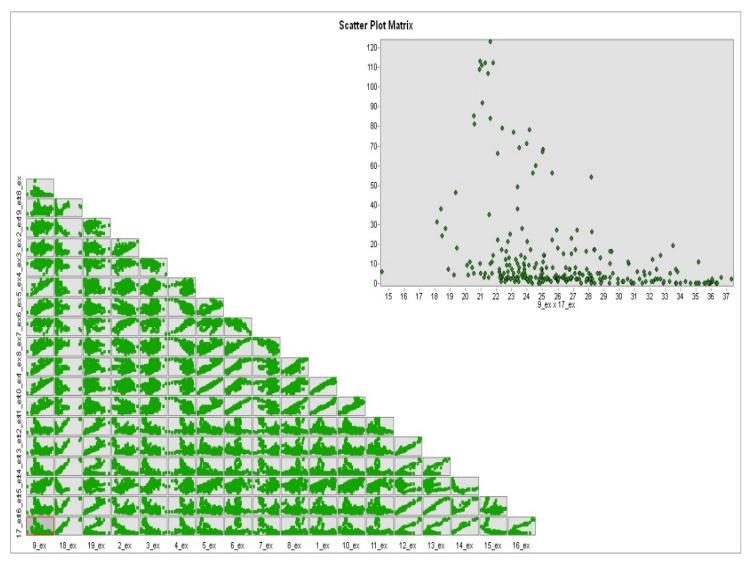
Scatter plot matrix for 19 bioclimatic variables.

In order to identify the main factors of bioclimate, PCA was applied. PCA (Principal Component Analysis) shows 11 bioclimatic variables that have autocorrelation (Figure 2); therefore, they were excluded from the study.

Box 2 presented eight bioclimatic variables, and these are included in this study.

Box 2 **Included bioclimatic variables.**

BIO1 = Annual Mean Temperature

BIO2 = Mean Diurnal Range (Mean of monthly (max temp–min temp))

BIO4 = Temperature Seasonality (standard deviation × 100)

BIO8 = Mean Temperature of Wettest Quarter

BIO12 = Annual Precipitation

BIO14 = Precipitation of Driest Month

BIO15 = Precipitation Seasonality (Coefficient of Variation)

BIO19 = Precipitation of Coldest Quarter

**Figure 3:**
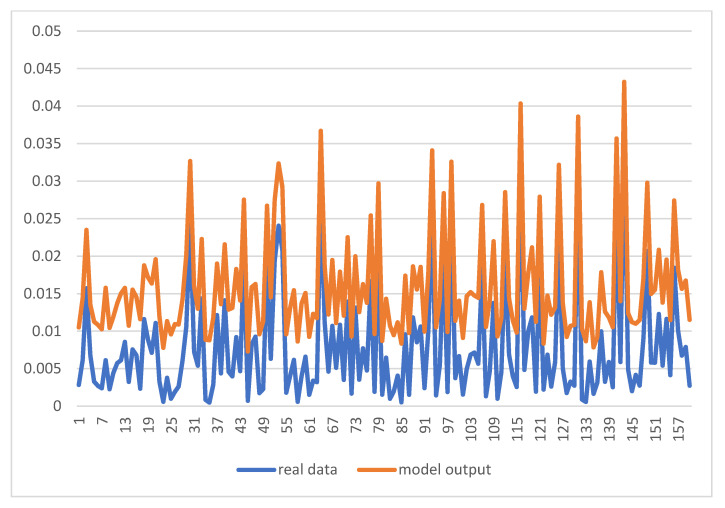
Real data and model output of SARS infection in 169 cities in Iran.

After the normalization and exclusion of outliers, the best input for the GFF’s (Generalized Feed Forward) model was identified, which were bio 12, bio 2, and bio 14. The GFNN’s (generalized feed-forward type of neural network) model predicted SARS infection per population in each city of Iran. Firstly, the difference between the real and model outputs was acceptable (based on MSE (Mean Square Error) and MAE (Mean Absolute Error)) (Figure 3).

**Figure 4:**
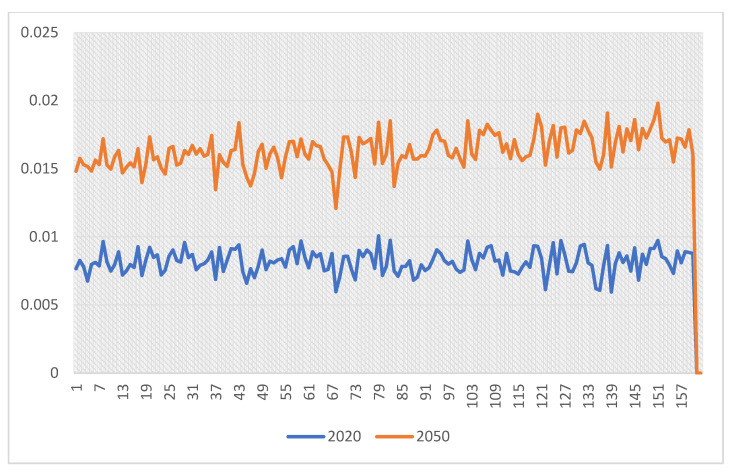
Normalized number of SARS infection in 169 cities of Iran in 2020 and 2050 (based on the RCP4.5 scenario in 2050).

Then, by means of the fitted model, the normalized number of SARS-infected cases in cities of Iran was obtained based on the RCP4.5 scenario in 2050 (Figure 4). Since the input values were normalized, the output values of the model were also normal. There was a statistically significant difference in the percentage of patients per population when cities of Iran were compared in 2050 according to the RCP4.5 scenario and the current values. The results have shown the SARS prevalence will be increased by 36% in some cities.

According to the obtained results, changes in bioclimatic parameters, the number of SARS patients will also be increased in cities of Iran. The highest increase was observed in Abadan city (Khuzestan province), Nehbandan city (South Khorasan province), Shahrereza (Isfahan province), and Shadegan (Khuzestan province). In addition, regarding climate change prediction in 2050, the highest number of SARS patients per population of each city was observed in Mehriz city (Yazd province), Abadeh city (Fars province), Zarand city (Kerman province), and Neyriz city (Fars province), respectively, based on estimated simulations.

## 4. Discussion

Respiratory diseases are a well-known outcome of climate change. Based on evidence obtained in this study, respiratory diseases such as SARS are a consequence of climate change in Iran. Scientific research shows that seasonal changes in temperature, absolute humidity (AH), sunlight, vitamin status, and host behavior affect the distribution of respiratory infection. These proposed factors can be classified as seasonal changes in the environment, human behavioral patterns, and viral factors. Environmental factors influence host susceptibility by modulating airway defense mechanisms as well as the viability and transmission of respiratory viruses. Human behavioral patterns also affect the contact rates between infected individuals and susceptible ones [36]. In line with previous studies, our findings showed a relationship between the ambient temperature and SARS infection [21]. Anderson and colleagues (2013) found, in the US, an increase in daily temperature by 10 F leads to an increase in hospitalization of individuals with respiratory infection by 4.3%, which is consistent with our results [23]. Consistent with this study, Silva et al. (2014) in Brazil, Zhang et al. (2019) in China, Pudpoung et al. (2011) in Thailand, Alessandrini et al. (2011) in Italy, and Wichmann et al. (2011) in Denmark have shown that temperature has a significant association with SARS infection [25,27,28,37]. In agreement with our results, one study in New Zealand demonstrated that the SARS infection rate is increased on unusual cold and warm days [26]. In Iran, according to the obtained findings, an increase in the minimum temperature in a semi-arid cold climate and a reduction in the maximum temperature in an arid-hot climate result in the increase in SARS infection.

Moreover, this study found the incidence of SARS will increase by 36% by 2050 in some cities of Iran. In line with this finding, one study conducted in New York estimated that excess respiratory admissions in NYS due to excessive heat would be 2 to 6 times higher in 2080–2099 than in 1991–2004. Bambrick et al. (2008) predicted that, relative to 1990, the total annual number of temperature-related hospital admissions in Australia would be increased 185–186% by 2050 and 217–223% by 2100, which is higher than the 4.5% increase that we projected for respiratory diseases in Iran (in total) based on the RCP 4.5 scenario in 2020–2050 [38]. Furthermore, Peng et al. (2011) predicted 166–2217 deaths/year in Chicago in 2081–2100 as a result of heatwaves using the A2, A1B, and B1 climate change scenarios. A study performed on the basis of the climate scenario estimated a 253% increase in annual heat-related mortality in the United Kingdom until the 2050 s under the medium-high UKCIP scenario for the temperature increase [39]. According to the range of SRES scenarios (A1, A2, B1, B2), Dessai et al. (2003) estimated that the annual heat-related deaths in Lisbon, Portugal, will increase from 5.4–6 per 100,000 for 1980–1998 to 7.3–35.6 per 100,000 by the 2050 s [40].

In the present study, the prediction of future SARS infection on the basis of the Mean Diurnal Range [Mean of monthly (max temp–min temp)] and Annual Precipitation greatly varies across the 169 cities in Iran. We found that southern regions, such as Khuzestan province, South Khorasan province, and Isfahan province, would have a larger proportional increase in SARS based on the RCP 4.5 scenario than other regions. This finding is consistent with a study carried out by Baaghideh and colleagues (2017), who indicated that an increase in temperature in the next decades in Mashhad and a 1 °C increase in maximum temperature are associated with a 4.27% (95%CI: 0.91, 7.00) increase in the mortality rate of cardiovascular disorders.

The present study has several advantages. It is one of the first studies specifically evaluating SARS infection and its related climate variables. These outcomes may be useful metrics for public health policymakers involved in planning for potentially increased public health and economic burdens resulting from global warming in the future. The assessment of current and future public health problems due to respiratory diseases would be significant since COVID-19 continues to increase in Iran. Studies show that SARS-CoV (causative pathogen for Severe Acute Respiratory Syndrome or SARS) and SARS-CoV-2 (causative pathogen for Coronavirus Disease 2019 or COVID-19) are positive-sense RNA viruses belonging to the family of Coronaviridae that are capable of causing severe respiratory diseases.

However, our findings need to be interpreted cautiously because of inherent uncertainties in our methods. First, our projections assume that associations between bioclimatic variables and SARS infection remain constant over time, which does not account for possible physiological and behavioral adaptation to climate change.

One important uncertainty is that we assumed that the size and demographic characteristics of each regional population remain constant at baseline levels in our projections of future health impacts. The increase in the population of cities, as well as the number of vulnerable groups, can increase the number of respiratory patients in the future, as the sensitivity analyses in other studies have shown the incidence of infection is higher among children, the elderly, and lower-income groups. The most important limitation of this study was the lack of long-term data on SARS infection that limited the information to only three decades. Therefore, we were not able to study the effects of climate change on SARS in the past decades (for instance, the last half-century).

The most important limitation of this study was the lack of long-term SARS data that led to the observation period being limited to only three decades. Therefore, we could not study the effects of climate change on SARS in the past decades (for example, the last half century) very well.

## 5. Conclusions

Our study shows a significant relationship between SARS and the climatic variables by the type of climate and gender, and the estimates suggest that hospital admissions for climate-related respiratory diseases in Iran will be increased by 36% in 2020–2050. This study demonstrates one of the health impacts of climate change. Policymakers can control the risks of climate change by mitigation and adaptation strategists. This study provides evidence for policy papers in the health sector.

## Figures and Tables

**Table 1:** Distribution of climate zones and in Iran. Base: 2000 and SARS distribution 2016–2018. Reprinted from: The relationship between dysentery and climatic parameters in Iran, vol. 34, Giti Bahrami, Mehdi Noroozi, Alireza Shakiba, Hassan Rafiey, HomeiraSajjadi, Pages No.: 100,697, Copyright (2022), with permission from Elsevier [32] (except the column of percentage of SARS distribution).

Description of Climate Type in Iran Name of Climate Type	Type of Climate Based on Köppen	Code of Climate in This Study (Based of Köppen)	Percentage of This Climate Type in Iran Base Classification in 2000	Percentage of SARS Distribution in Iran between 2016–2018
Semi-arid warm	BSh	211	14.24	24.85
Semi-arid cold	BSk	212	12.82	22.15
Arid-hot	BWh	221	43.30	12.23
Arid-cold	BWk	222	2.58	6.76
Humid subtropical	Cfa	311	0.40	2.87
Subtropical with arid summer	Csa	331	5.36	5.22
Continental temperate	Dca	411	0.06	-
Arid-warm and temperate	Dcsa	421	8.01	11.34
Arid-cold and temperate	Dcsb	422	5.10	0.99
Humid, hot and temperate oceanic	Doa	451	4.33	9.19
Arid-warm temperate with a winter precipitation regime	Dcwa	431	1.95	4.04
Arid-cold temperate with a winter precipitation regime	Dcwb	432	1.22	-

**Table 2:** Summary of meteorological and SARS information used in the present study.

Time Span	Sampling Frequency	Variable
17 February 2016–17 February 2018	daily	Maximum temperature
17 February 2016–17 February 2018	daily	Minimum temperature
17 February 2016–17 February 2018	daily	Precipitation
17 February 2016–17 February 2018	daily	Incidence of SRRS In women
17 February 2016–17 February 2018	daily	

**Table 3:** MAE and RMSE for predicting SARS model.

Model Statistics	Exper.	Project
Data Set	Optim.	Leave-N-Out
Score	60/172	42/512
Avg. Correlation	0/340	0/000
Avg. Norm. MSE	0/233	0/278
Avg. Norm. MAE	0/172	0/206
Max. Abs. Error	0/026	0/030
Training Epochs	57	
Training Seconds	1	

**Table 4:** The relationship between SARS and climatic factors by gender in Iran.

Variable	Women			Men		
	IRR	*p* Value	95% CI	IRR	*p* Value	95% CI
t min °C	0.99	0.169	0.98–1.00	0.98	<0.000	0.98–0.99
t max °C	1.00	0.716	0.99–1.00	1.00	0.549	0.99–1.00
rrr24 ^a^ mL	1.00	0.539	0.99–1.00	0.99	0.060	0.98–1.00

^a^ RRR24: Amount of *precipitation* accumulated during the preceding 24 h.

**Table 5:** Relationship between SARS and climate factors by gender and climate in Iran.

Type of Climate	Variable	Women			Men		
		IRR	*p* Value	95% CI	IRR	*p* Value	95% CI
Csa	t max	1.00	0.373	0.98–1.03	1.02	0.075	0.99–1.04
BWk	t min	1.01	0.117	0.99–1.04	1.01	0.127	0.99–1.03
BWk	rrr24	0.74	0.075	0.53–1.03	0.61	0.030	0.39–0.95
BSk	t min	1.01	0.004	1.00–1.02	1.01	0.010	1.00–1.03
BSk	rrr24	1.02	0.000	1.00–1.05	1.03	0.003	1.01–1.05
BWh	T min	0.98	0.136	0.97–1.00	0.97	0.005	0.95–0.99
BWh	T max	0.98	0.046	0.97–0.99	0.99	0.289	0.97–1.00
BSh	t min	0.98	0.059	0.97–1.00	0.98	0.001	0.97–0.99
Dcsa	t min	1.01	0.126	0.99–1.03	1.02	0.071	0.99–1.04
Dcsa	t max	1.00	0.257	0.99–1.02	0.98	0.078	0.96–1.00
Doa	t max	0.98	0.038	0.96–0.99	0.96	<0.000	0.95–0.98
Doa	rrr24	0.99	0.642	0.97–1.01	0.95	0.009	0.92–0.98
Dcwa	t min	0.98	0.261	0.95–1.01	0.95	0.001	0.93–0.98
Dcwa	t max	0.98	0.381	0.95–1.01	0.99	0.973	0.97–1.02
Dcwa	rrr24	1.00	0.901	0.96–1.03	0.97	0.286	0.92–1.02

## Data Availability

Data available on request due to legal restrictions. The data presented in this study are available on request from the corresponding author. The data are not publicly available.
